# Plant Species Loss Affects Life-History Traits of Aphids and Their Parasitoids

**DOI:** 10.1371/journal.pone.0012053

**Published:** 2010-08-06

**Authors:** Jana S. Petermann, Christine B. Müller, Christiane Roscher, Alexandra Weigelt, Wolfgang W. Weisser, Bernhard Schmid

**Affiliations:** 1 Institute of Evolutionary Biology and Environmental Studies, University of Zurich, Zurich, Switzerland; 2 Max Planck Institute for Biogeochemistry, Jena, Germany; 3 Institute of Ecology, University of Jena, Jena, Germany; University of Plymouth, United Kingdom

## Abstract

The consequences of plant species loss are rarely assessed in a multi-trophic context and especially effects on life-history traits of organisms at higher trophic levels have remained largely unstudied. We used a grassland biodiversity experiment and measured the effects of two components of plant diversity, plant species richness and the presence of nitrogen-fixing legumes, on several life-history traits of naturally colonizing aphids and their primary and secondary parasitoids in the field. We found that, irrespective of aphid species identity, the proportion of winged aphid morphs decreased with increasing plant species richness, which was correlated with decreasing host plant biomass. Similarly, emergence proportions of parasitoids decreased with increasing plant species richness. Both, emergence proportions and proportions of female parasitoids were lower in plots with legumes, where host plants had increased nitrogen concentrations. This effect of legume presence could indicate that aphids were better defended against parasitoids in high-nitrogen environments. Body mass of emerged individuals of the two most abundant primary parasitoid species was, however, higher in plots with legumes, suggesting that once parasitoids could overcome aphid defenses, they could profit from larger or more nutritious hosts. Our study demonstrates that cascading effects of plant species loss on higher trophic levels such as aphids, parasitoids and secondary parasitoids begin with changed life-history traits of these insects. Thus, life-history traits of organisms at higher trophic levels may be useful indicators of bottom-up effects of plant diversity on the biodiversity of consumers.

## Introduction

The consequences of the ongoing loss of plant species have been studied intensively, but rarely at other trophic levels than that of the plants themselves [1], [2]. If plant species disappear from a multi-trophic system, it is conceivable that the effects of this loss of primary producers “cascade up” the system and cause changes at higher trophic levels. The first bottom-up cascades [3] were described from aquatic systems, but they have since been suggested to operate in a similar way in terrestrial systems [4]. Indeed, a few field and laboratory experiments have demonstrated terrestrial “community- and population-level” bottom-up cascades [4], such as effects of plant abundance and richness on the abundance and richness of herbivores or predators [5], [6], [7], [8], [9], [10], [11]. In contrast to these community- and population-level approaches, other studies examined cascading effects at the level of the individual organism (“individual-level cascades” [4]). These studies typically manipulated plant quality and measured effects on life-history traits of individuals at higher trophic levels [12], [13], [14], [15], [16], [17]. In this study, we hypothesize that changes in plant community diversity affect life-history traits of insects at higher trophic levels via changes in plant abundance and quality, effectively linking community- and individual-level cascades.

To address this hypothesis, we used aphid–parasitoid communities as a model system. The advantage of this system is that both aphids and their parasitic wasps have a relatively short range of movement, and due to their high degree of specialization are strongly dependent on the presence and quality of their host species [18], [19], [20], [21]. Aphids often prefer single or few host plant species at least during part of their life cycle [18]. Once a winged (alate) aphid colonizer has reached such a host plant, a colony forms via asexual reproduction of non-winged (apterous) individuals. These remain rather immobile on the same plant individual. Wasps parasitize aphids by laying single eggs into their body [22]. The parasitoid larva feeds internally on the aphid until the aphid dies and forms a hard-shelled “mummy” in which the parasitoid larva pupates. The adult primary parasitoid emerges from the mummy shortly after, unless it is itself parasitized by a secondary parasitoid. These secondary parasitoids attack the primary parasitoid either while the aphid is still alive (koinobiont “hyperparasitoids”) or when the aphid has developed into a mummy (idiobiont “mummy parasitoids”).

In this study, we recorded four life-history traits of aphids and their primary and secondary parasitoids that we expected to vary along a plant richness gradient: (1) the proportion of winged individuals in aphid colonies, (2) the emergence proportion of parasitoids, (3) the proportion of parasitoids that are female and (4) the body mass of the two most abundant parasitoids. Wings are a costly trait for aphids that has been associated with reduced fecundity and are therefore not constantly expressed [18], [23]. The production of winged morphs in aphids has been suggested to serve two main purposes besides the obligatory change of host plant species in the life cycles of some species: firstly, the avoidance of deteriorating nutritional conditions, and secondly, the escape from predators [23], [24], [25], [26]. While wing production is subject to intra- and interspecific variation [23], the proportion of winged aphids in a colony may reflect its nutritional or enemy environment which we anticipate to change with changing plant diversity.

Whether a parasitoid larva develops and emerges successfully from the aphid mummy as an adult depends amongst other things on aphid quality [27] and resistance [28], [29]. Therefore, if changes in the plant community affect the aphids' quality in terms of nutritional value, size or defensive abilities, these changes are expected to influence the proportion of successfully emerging parasitoids.

Parasitoid body mass is generally correlated with fitness [30], [31], which has been shown to be controlled by host nutritional quality and especially by host size [e.g. 21], [32]. Furthermore, some hymenopteran parasitoids are able to select the sex of their offspring at oviposition: fertilized eggs develop into females, unfertilized ones into males [30]. Because female parasitoids gain more from being large [33], [34], female eggs are typically laid into the highest-quality hosts [33]. Thus, heavier parasitoids with a female-biased sex ratio could be expected to emerge in favorable environments.

In this study, we measured these life-history variables of aphids and parasitoids in experimental grassland communities varying in plant species richness and legume presence and show that (1) plant species loss has bottom-up cascading effects on life-history traits of organisms at higher trophic levels, (2) these cascades are mediated by diversity-related changes in host plant biomass and host plant nitrogen concentration.

## Methods

### Experimental design

This study was conducted as part of the Jena Experiment, a temperate grassland biodiversity experiment in Jena, Germany [35]. The experimental site is a floodplain area close to the river Saale, which was used for agricultural cropping before the experiment started in 2002. The site has not received fertilizer since then and is mown twice per year, a typical mowing regime of these grasslands. In the 3.5×3.5 m plots that were used for this study, nine dominant plant species from semi-natural, mesophilic grasslands were sown in 2002 as monocultures and mixtures (richness levels of 1, 2, 3, 4, 6, and 9 species [36]). The original species compositions were maintained by weeding at regular intervals [35]. The plots were arranged in four blocks with increasing distance to the river. Preliminary surveys showed that aphids could be regularly found on four of the nine species at the field site (*Anthriscus sylvestris*, *Arrhenatherum elatius*, *Phleum pratense* and *Trifolium pratense*, plant nomenclature follows Rothmaler [37]). Hence, only plots containing at least one of these host plant species at a minimum abundance (>5% cover in May 2006) were used for this study (Table S1). These selection criteria resulted in an unbalanced design with fewer plots at the high plant-species richness end of the gradient. Therefore, we interpret our results with caution.

### Data collection

We identified and counted all aphids (a total of >16,000 individuals) that were part of an established colony on a host plant, including nymphs and winged morphs, in the same 0.2×3 m transect across the middle of the plots four times from May (first appearance of aphids at the field site) to August 2006 (hardly any aphids found after the fourth sampling period). All sampling campaigns were completed within about one week: two campaigns before the first mowing of the field site (around 2 June and 17 June) and two between the first and the second mowing (20 July and 2 August). Sampling was usually done in the mornings from a bench across the plots to avoid disturbing the vegetation and the aphid populations.

All parasitized aphids (mummies) encountered in the transects during sampling were collected. Additionally, all mummies in a surrounding plot area (usually 4 m^2^) were collected at the same time to be able to detect the full parasitoid community of a plot. A total of more than 3,500 mummies were collected, about one third of them from the transects. All mummies were placed individually in gelatin capsules in the field and were taken to the laboratory for rearing of the parasitoids. To induce parasitoid emergence, all mummies remaining after four months were subjected to a 3-month cold-warm-cold cycle with a minimum of 4°C. A total of about 2,000 parasitoids (about 1,700 primary parasitoids and 300 secondary parasitoids) emerged and were identified to species level. We individually weighed all 1,072 emerged individuals of the two most abundant parasitoid species (both primary parasitoid species) on a microbalance (Mettler Toledo MX5) to the nearest microgram after drying at 70°C for 2 days. Those two species were *Adialytus arvicola* (Stary), predominantly parasitizing the aphid *Sipha maydis* (aphid nomenclature follows Stresemann [38]) on the host plant *A. elatius*; and *Trioxys brevicornis* (Haliday), predominantly parasitizing the aphid *Cavariella aegopodii* on the host plant *A. sylvestris*.

The biomass of aphid host plants was determined by clipping the vegetation at a height of 3 cm in two 20×50 cm areas within each plot in May and August 2006. The harvested biomass was sorted into species, dried at 70°C for 48 h and weighed. The biomass data were averaged over the two sampling areas and summed up over the whole year. Nitrogen concentrations of aphid host plants were measured for each plant species from biomass samples harvested in May 2004 [39] in the same plots that were used for aphid counts or in replicate plots with the same plant composition.

### Data analysis

The proportion of winged aphid morphs was calculated separately for each aphid species by dividing the density of winged individuals by the total density of this species. Alatiform nymphs were rarely encountered and included as non-winged morphs; parasitized aphids were excluded. The proportion of winged aphids was arcsine square-root transformed to improve normality and homoscedasticity. The emergence proportion of parasitoids was calculated as the number of emerged parasitoids divided by the sum of emerged parasitoids and remaining mummies for each aphid species. This analysis was done at the level of the aphid species because in the case of failed emergence we could not confidently distinguish between primary and secondary parasitoids or identify their species. The proportion of female parasitoids was calculated for each parasitoid species by dividing the number of female individuals by the total number of emerged individuals of this species. One parasitoid species, *Lysiphlebus fabarum* (Marshall) which parasitized the aphid *Aphis scaliai* on the legume *T. pratense*, reproduces predominantly asexually in Europe [40] and was excluded from the analysis of proportions of females. All data were aggregated over the four census times.

We used multiple regression and ANOVA to analyze the data. Except for the proportion of winged aphid morphs the response variables were not transformed for the final analyses because model-checking procedures indicated that this was not necessary and analyses with transformed variables gave similar results. The biomass of aphid host plants (in g/m^2^) and their nitrogen concentration (in %) were used as covariables. All analyses were carried out with the statistical software R, version 2.7.2 [41].

## Results

### Plant-related variables

Overall, host plant biomass declined with increasing plant species richness (Table 1). Host plant nitrogen concentration increased with increasing plant species richness and was higher in legume (2.3±0.8%) than in non-legume plots (1.4±0.3%) and higher for legume (*T. pratense*: 2.9±0.5%) than for non-legume host plants (*A. sylvestris*: 2.0±0.4%, *A. elatius*: 1.2±0.3%, *P. pratense*: 1.3±0.3%). Plant species on which mummies of the two most abundant parasitoid species (*A. arvicola* and *T. brevicornis*) were found, also showed a decrease in biomass with increasing plant species richness (F_1,24_ = 16.6, P<0.001). Their average biomass was higher in plots containing legumes (452±246 g/m^2^) than in plots from which legumes were absent (198±123 g/m^2^, F_1,24_ = 34.7, P<0.001). However, their nitrogen concentration did not significantly increase with plant species richness (F_1,18_ = 1.15, P = 0.297).

**Table 1:** ANOVAs for host plant biomass and host plant nitrogen concentration.

	Host plant biomass					Host plant nitrogen concentration				
	df	MS	F	P		df	MS	F	P	
Plant species richness	1	706360	17.3	**0.0001**	**↓**	1	2.21	4.1	**0.0482**	↑
Legume presence	1	3422	0.1	0.7735		1	16.23	30.3	**<0.0001**	↑
Plotcode	44	40806	4.8	**<0.0001**		43	0.54	23.3	**<0.0001**	
Host plant species (non-legume vs. legume)	1	488148	57.0	**<0.0001**	**↓**	1	32.37	1407.3	**<0.0001**	↑
Host plant species (rest)	2	13823	1.6	0.2061		2	0.78	34.0	**<0.0001**	
Plant species richness×Host plant species (non-legume vs. legume)	1	261584	30.6	**<0.0001**		1	0.01	0.3	0.5832	
Plant species richness×Host plant species (rest)	2	50123	5.9	**0.0044**		2	0.01	0.3	0.7387	
Legume presence×Host plant species (rest)	2	19150	2.2	0.1142		1	0.03	1.2	0.2743	
Residuals	71	8558				60	0.02			

The term representing host plant species identity (“Host plant species”) is split into two contrasts: “non-legume vs. legume” and the term containing the remaining variance from host plant species identity (“rest”). So for example (row 4), non-legume host plant species have a higher biomass than legume host plant species but other host plant species identity effects are not significant. “Plant species richness” and “Legume presence” were tested against “Plotcode”, all other terms against “Residuals”. Directions of significant main effects (with 1 df) are indicated by arrows. P-values<0.05 are printed in bold. MS = mean square.

### Proportion of winged aphids

**Figure 1:**
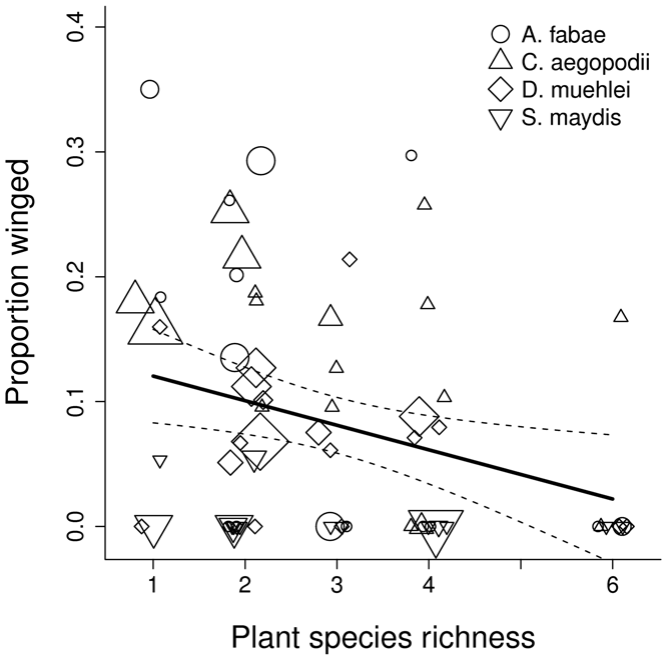
Proportion of winged aphids as a function of plant species richness. Only four aphid species produced winged morphs and are shown here: *A. fabae* (circles), *C. aegopodii* (up-facing triangles, both species mainly on the host plant species *A. sylvestris*) and *D. muehlei* (diamonds, on the host plant species *P. pratense*) and *S. maydis* (down-facing triangles, on the host plant species *A. elatius*). The size of the plotting symbol is proportional to the biomass (g/m^2^) of the host plant species of the respective aphid species in the respective plot. Host plant biomass below 200 g/m^2^ is represented by a symbol with a fixed minimal size. Proportions of winged aphids are arcsine square-root transformed. 95% confidence intervals are shown around the regression line.

Winged individuals were found in colonies of only four out of the ten aphid species (Table S2): *Aphis fabae*, *Cavariella aegopodi* (both mainly on the host plant *A. sylvestris*), *Diuraphis muehlei* (on the host plant *P. pratense*) and *Sipha maydis* (on the host plant *A. elatius*). The proportion of winged morphs declined with increasing plant species richness, both for these four aphid species (Fig. 1) and when all aphid species were included in the analysis (Table 2). The biomass of the aphid host plants explained part of this effect: higher host plant biomass had an increasing effect on the proportion of winged aphids, but since host plant biomass declined with plant species richness, the proportion of winged individuals declined as well. Aphid load (aphid density per host plant biomass, F_1,28_ = 0.083, P = 0.775) did not have a significant effect on the proportions of winged aphids. Parasitoid density in the plots had a positive effect on the proportions of winged aphids (F_1,28_ = 12.654, P = 0.001, respectively).

**Table 2:** ANOVAs for the proportion of winged aphids.

	a) without covariables				b) with covariables				
	df	%SS	F	P	df	%SS	F	P	
Host plant biomass					1	13.2	29.6	**<0.0001**	**↑**
Host plant nitrogen concentration					1	0.1	0.2	0.6902	
Plant species richness	1	7.0	7.1	**0.0111**	1	4.1	3.9	0.0546	
Legume presence	1	0.0	0.0	0.9595	1	0.1	0.1	0.7865	
Plotcode	42	41.9	2.1	**0.0097**	41	42.5	2.3	**0.0082**	
Aphid identity	9	27.4	6.3	**<0.0001**	9	23.2	5.8	**0.0001**	
Plant species richness×Aphid identity	6	2.1	0.7	0.6386	6	2.4	0.9	0.4996	
Legume presence×Aphid identity	4	0.9	0.5	0.7436	3	0.7	0.5	0.6687	
Residuals	43	20.7			31	13.8			

Proportions were arcsine square-root transformed prior to analyses. The first analysis (a) was done without covariables, the second analysis (b) with biomass and nitrogen concentration of aphid host plants as covariables. “Plant species richness” and “Legume presence” were tested against “Plotcode”, all other terms against “Residuals”. Directions of significant effects of the covariables are indicated by arrows. P-values<0.05 are printed in bold. % SS = percent sum of squares explained.

### Parasitoid emergence

**Figure 2:**
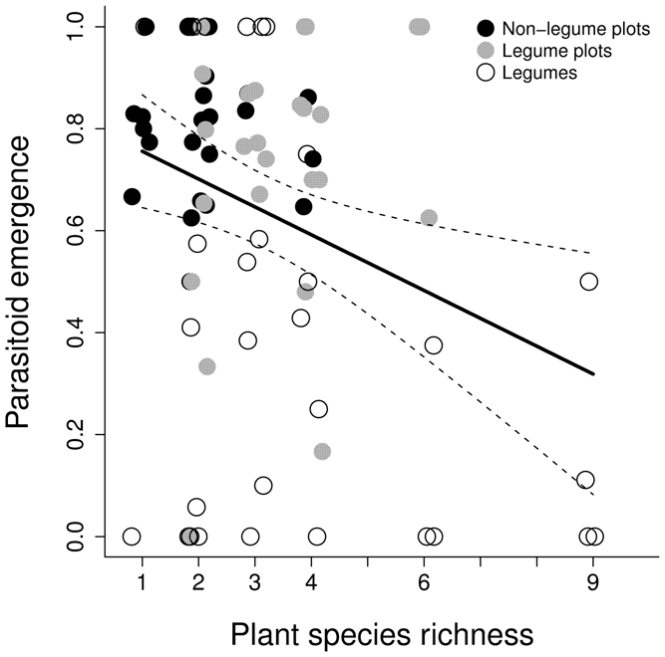
Parasitoid emergence proportion as a function of plant species richness. Closed black symbols depict parasitoids emerged from mummies on non-legume host plants (*A. sylvestris*, *A. elatius*, *P. pratense*) growing in plant communities without legumes, grey symbols depict parasitoids emerged from mummies on those non-legume host plant species in plant communities with legumes and open symbols depict parasitoids emerged from mummies on a legume host plant (*T. pratense*). The regression line (shown with 95% confidence intervals) was fitted to all data points.

The proportion of emerged parasitoids declined with increasing plant species richness. Furthermore, the proportion was lower in plots containing legumes. However, the latter was largely due to the fact that parasitoids of aphid species with legume host plants showed very low emergence proportions (Fig. 2, Table 3). Host plant nitrogen concentration had a significant negative effect on emergence (Table 3). When considering only aphid species that were hosts for the two most abundant parasitoids, *A. arvicola* and *T. brevicornis* (mummies only on non-legume host plants), emergence proportions were similar in legume and non-legume plots (F_1,25_ = 0.029, P = 0.867).

**Table 3:** ANOVAs for the parasitoid emergence proportion.

	a) without covariables				b) with covariables				
	df	%SS	F	P	df	%SS	F	P	
Host plant biomass					1	1.4	1.4	0.2591	
Host plant nitrogen concentration					1	18.6	17.6	**0.0007**	↓
Plant species richness	1	8.9	10.4	**0.0024**	1	5.9	7.4	**0.0092**	
Legume presence	1	5.5	6.4	**0.0149**	1	0.0	0.1	0.8075	
Plotcode	44	37.7	1.2	0.3621	43	33.9	0.7	0.7818	
Aphid identity	6	23.6	5.3	**0.0014**	6	16.2	2.6	0.0629	
Plant species richness×Aphid identity	4	4.9	1.7	0.1941	4	5.4	1.3	0.3198	
Legume presence×Aphid identity	2	1.6	1.1	0.3523	1	1.6	1.5	0.2433	
Residuals	24	17.8			16	16.9			

The first analysis (a) was done without covariables, the second analysis (b) with biomass and nitrogen concentration of aphid host plants as covariables. “Plant species richness” and “Legume presence” were tested against “Plotcode”, all other terms against “Residuals”. Directions of significant effects of the covariables are indicated by arrows. P-values<0.05 are printed in bold. % SS = percent sum of squares explained.

### Proportion of female parasitoids

**Figure 3:**
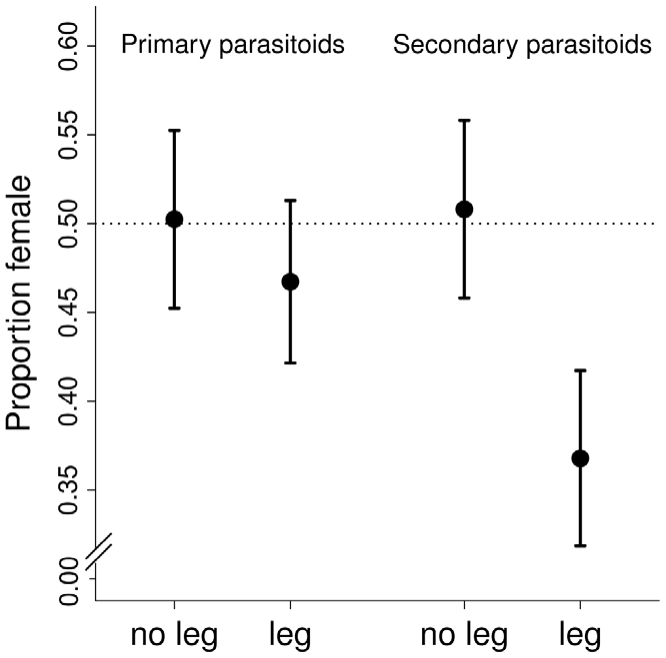
Proportion of female primary and secondary parasitoids in communities with and without legumes. The dashed line shows the 1∶1 sex ratio.

The proportion of female parasitoids was lower in the presence of legumes, irrespective of the trophic level or species identity of the parasitoid (Fig. 3, Table 4). Host plant biomass had a significant negative effect on the proportion of female parasitoids and explained part of the negative legume effect (Table 4). There was no significant legume effect for the two most abundant parasitoids, *A. arvicola* and *T. brevicornis* (F_1,24_ = 0.528, P = 0.475).

**Table 4:** ANOVAs for the proportion of female parasitoids.

	a) without covariables				b) with covariables				
	df	%SS	F	P	df	%SS	F	P	
Host plant biomass					1	3.6	6.5	**0.0148**	↓
Host plant nitrogen concentration					1	1.6	2.9	0.0988	
Plant richness	1	0.7	0.9	0.3461	1	0.8	0.9	0.3432	
Legume presence	1	3.3	4.3	**0.0452**	1	0.3	0.3	0.5732	
Plotcode	39	30.1	1.6	0.0555	37	30.9	1.5	0.1081	
Trophic level	1	0.0	0.0	0.9736	1	0.4	0.8	0.3735	
Parasitoid identity	18	20.5	2.3	**0.0077**	18	22.3	2.2	**0.0191**	
Plant species richness×Trophic level	1	0.0	0.0	0.9086	1	0.1	0.1	0.7182	
Plant species richness×Parasitoid identity	15	7.1	1.0	0.5051	14	10.1	1.3	0.2496	
Legume presence×Trophic level	1	0.1	0.1	0.7156	1	0.2	0.4	0.5296	
Legume presence×Parasitoid identity	12	7.9	1.3	0.2216	12	9.2	1.4	0.2157	
Residuals	62	30.4			37	20.5			

The first analysis (a) was done without covariables, the second analysis (b) with biomass and nitrogen concentration of aphid host plants as covariables. “Plant species richness” and “Legume presence” were tested against “Plotcode”, all other terms against “Residuals”. “Trophic level” signifies the contrast between primary and secondary parasitoids. Directions of significant effects of the covariables are indicated by arrows. P-values<0.05 are printed in bold. % SS = percent sum of squares explained.

### Parasitoid body mass

**Figure 4:**
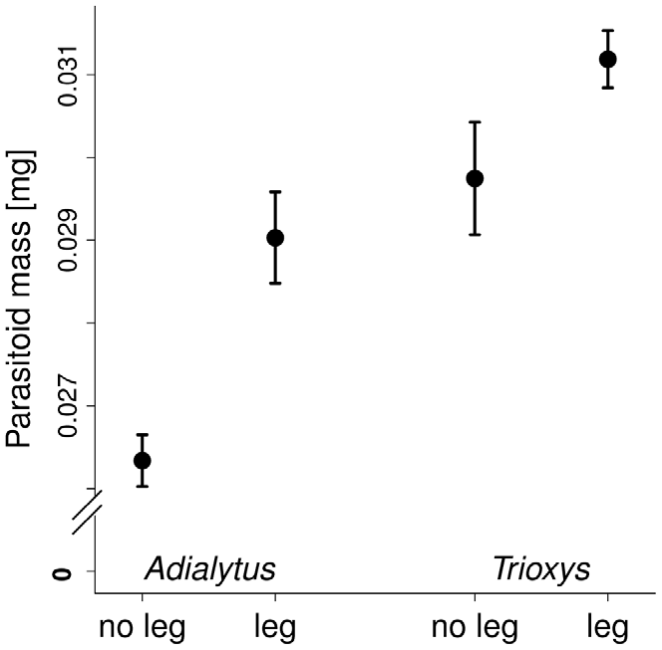
Body mass of two primary parasitoids in plant communities with and without legumes. Average dry mass of the two most abundant primary parasitoids *A. arvicola* and *T. brevicornis* ± standard error (mg) in plots without legumes (“no leg”) and with legumes (“leg”). Both parasitoid species developed only in aphids on non-legume host plant species (*A. elatius* and *A. sylvestris*, respectively).

The body mass of the two most abundant parasitoids, *A. arvicola* and *T. brevicornis* was similar across all plant species richness levels but was higher in the presence of legumes for males and females of both parasitoid species (Figs. 4 and S1, Table 5). Because both species emerged only from mummies on non-legume host plants, these effects were indirect: they were largely due to increased biomass and nitrogen concentration of these non-legume host plants in plots containing legumes.

**Table 5:** ANOVAs for the body mass of the two most abundant primary parasitoid species (*A. arvicola* and *T. brevicornis*).

	a) without covariables				b) with covariables				
	df	%SS	F	P	df	%SS	F	P	
Host plant biomass					1	6.1	67.1	**<0.0001**	↑
Host plant nitrogen concentration					1	2.5	27.4	**<0.0001**	↑
Plant species richness	1	0.3	1.4	0.2452	1	0.8	2.7	0.1228	
Legume presence	1	7.7	34.6	**<0.0001**	1	0.6	2.2	0.1584	
Parasitoid identity	1	1.7	7.7	**0.0103**	1	1.1	3.9	0.0664	
Plant species richness×Parasitoid identity	1	0.2	0.7	0.4190	1	0.1	0.5	0.5017	
Legume presence×Parasitoid identity	1	0.0	0.1	0.7088	1	0.2	0.8	0.3812	
Plotcode	24	5.3	2.6	**0.0001**	16	4.7	3.2	**<0.0001**	
Sex	1	2.8	32.8	**<0.0001**	1	2.4	26.3	**<0.0001**	
Plant species richness×Sex	1	0.1	1.7	0.1917	1	0.2	1.7	0.1894	
Legume presence×Sex	1	0.2	2.0	0.1551	1	0.2	2.3	0.1297	
Parasitoid identity×Sex	1	0.7	8.5	**0.0036**	1	0.5	5.9	**0.0158**	
Residuals	936	80.9			877	80.5			

The first analysis (a) was done without covariables, the second analysis (b) with biomass and nitrogen concentration of aphid host plants as covariables. Both parasitoid species developed only in aphids on non-legume host plant species. The covariables and “Sex” (incl. all interactions with “Sex”) were tested against “Residuals”, all other terms against “Plotcode”. “Parasitoid identity” was tested at the “Plotcode” level in this analysis because in almost all cases only one of the two species was present in a plot. Directions of significant effects of the covariables are indicated by arrows. P-values<0.05 are printed in bold. % SS = percent sum of squares explained.

## Discussion

Our results demonstrate that plant species richness and the presence of legumes in plant communities affect the life history of aphids and their parasitoids both directly and indirectly via host plant biomass and nitrogen concentration. These effects are paralleled by community-level cascading effects of plant species richness on species richness and densities of aphids and their parasitoids that we reported previously from the same field site [11].

### Proportion of winged aphids

The proportion of winged aphids decreased with increasing plant species richness, partly as a result of a simultaneous decrease in aphid host plant biomass. Whereas low host plant abundance typically has a negative effect on herbivore densities [5], [6], [11], [42], the proportion of winged individuals was expected to rise with resource limitation since wing production after the initial colonization period in early spring is a strategy to escape deteriorating conditions by dispersing [23], [25]. In addition to the unexpected direction of the effect, we found no influence of crowding (aphid load) or of plant quality (in terms of plant nitrogen concentration) on winged morph production, suggesting that limited resource availability was not the cause for decreasing proportions of winged morphs with increasing plant species richness. However, Züst et al. [43] suggested that resources might sometimes be too low to induce wing production at all. So the resource-limitation argument could be reversed to explain the extremely low proportions of winged aphids in communities with high plant species richness and low host plant biomass. Parasitoid density had an increasing effect on aphid wing production indicating that increased pressure from natural enemies might have played a role in this context. However, we found relevant proportions of winged individuals in only four aphid species. It has been shown that intra- and interspecific variation in wing induction can be very large [23]. Furthermore, other abiotic effects that we did not measure (e.g. temperature) may be important triggers [23], cautioning against drawing strong conclusions from the results.

### Parasitoid emergence and proportion of female parasitoids

Emergence proportions of parasitoids similarly decreased with increasing plant species richness. However, our design was unbalanced, because of a disproportionately low number of plant communities at high species richness levels. Therefore, we have to interpret our results with caution. Both emergence proportions and proportions of females were lower in plant communities containing legumes compared with legume-free communities. These relationships were partially explained by changes in host-plant nitrogen concentration (parasitoid emergence) and in host plant biomass (proportion of females).

Plant nitrogen concentrations, which are often increased even in non-legumes by the presence of nitrogen-fixing legumes [44], [45], can have positive or negative effects on higher trophic levels. On the one hand, plants with higher nitrogen concentrations may be more nutritious for herbivores, which are generally nitrogen-limited [46], [47], [48], [49]. On the other hand, nutrient availability is known to affect plant allocation to secondary metabolites [50], [51] and plants may use surplus nitrogen to produce higher levels of nitrogen-based defense compounds [51], [52]. One trophic level above herbivores, the same dichotomy may become apparent. Natural enemies of herbivores are even more strongly limited by nitrogen than the herbivores themselves [53], [54] and might do better on nitrogen-rich hosts. However, some herbivores can use nitrogen-based plant compounds or simply their own high nutritional status on nitrogen-rich host plants to mount defenses against predators and parasitoids [55], [56]. While some of the early defense reactions (encapsulation and abortion of parasitoid eggs) probably went unnoticed in our study, the lower parasitoid emergence success on nitrogen-rich host plants could still be due to increased aphid defenses.

Since mated parasitoid females can determine the sex of their offspring at oviposition [30], we speculate that they might have sensed a potentially different host quality and avoided “wasting” females on risky hosts. On the other hand, the parasitoid sexes might have differential mortality [30], with female larvae more prone to abortion as a result of lower host quality, potentially explaining the lower emergence success in legume plots. Interestingly, even secondary parasitoids showed the effect of legumes on the proportions of females, implying a cascade to the third trophic level above the plants.

### Parasitoid body mass

In contrast to general parasitoid emergence and proportions of females, the body mass of the two most abundant primary parasitoids, *A. arvicola* and *T. brevicornis*, was higher in the presence of legumes in the plant community. Host plant nitrogen concentration and host plant biomass had a positive effect in this case. Since we did not find a negative effect of legume presence on emergence or the proportion of females in these two species, we conclude that they might be resistant to potentially increased aphid defenses in legume plots. This resistance may even explain their high abundance at our field site. The two species seemed to instead profit from the presence of legumes. We did not measure aphid body mass or nitrogen content and hence have no conclusive evidence for either higher nitrogen content of hosts [53], [54], [57] or larger host size indeed mediating higher parasitoid body mass in communities with legumes. Another field experiment found a higher body-mass gain of herbivores (grasshoppers) in legume-containing plant communities [58]. Since host size is typically strongly correlated with parasitoid size [59], a size-mediated effect seems plausible.

### Conclusions

We found considerable effects of plant species richness and community composition cascading up the food web to affect aphid and parasitoid life-history traits. Legumes exerted a particularly strong effect in some cases. This plant functional group had an impact up to the highest trophic level considered in this study, sometimes mediated via an additional interaction at the plant level, thus affecting even insect communities on non-legume host plants.

In contrast, we found only plant-species richness effects but no effects of legume presence on aphid and parasitoid richness and densities in a community-level study at the same field site [11], underlining the importance of life-history studies to detect different pathways of effects of plant species loss on associated biodiversity. This difference in the effect of legume presence between the two studies may indicate that cascading bottom-up effects on insect life-history traits are more strongly resource-quality mediated, whereas cascading effects on insect species richness and density seem to be rather mediated by abundance and richness of lower trophic levels. On the other hand, it is possible that insect life-history studies detect effects of plant species loss at an earlier stage than richness or abundance studies. Similar observations have been made in analyses of vegetation responses to habitat change, where life-history changes could be used as early indicators [60].

The altered life-history traits of aphids and parasitoids are expected to eventually result in altered species abundances and richness at these consumer trophic levels. One example for this link is the apparently low suitability of species-rich plant communities for parasitoids. In the present study, we found for example lower emergence success, potentially due to increased aphid defenses. These are paralleled by low parasitoid densities and species richness [11], possibly a result of reduced parasitoid reproduction. We therefore advocate the additional examination of individual-level cascades to predict and explain population- and community-level cascades of plant species loss on higher trophic levels.

## Supporting Information

Figure S1 Body mass of two primary parasitoids in communities with and without legumes. Average dry mass of a) females and b) males of the two most abundant primary parasitoids *A. arvicola* and *T. brevicornis* ± standard error (mg) in plots without legumes (“no leg”) and with legumes (“leg”). Both parasitoid species developed only in aphids on non-legume host plant species (*A. elatius* and *A. sylvestris*, respectively).
(0.06 MB TIF)

Table S1 List of all 47 sampled plots. For each plot, block number, plot number, plant species richness, the presence of legumes, the presence of the nine plant species in the plant assemblage and the total host plant biomass (sum across all aphid host plant species and both harvests in 2006) are given. In some cases, two replicate plots with the same plant composition were used. The presence of a plant species refers to its presence in the seed mixture at plot establishment in 2002, independent of its actual abundance through 2006. Only the first four plant species hosted aphids in our study. *Ant: Anthriscus sylvestris, Arr: Arrhenatherum elatius, Phl: Phleum pratense, Tri p: Trifolium pratense, Alo: Alopecurus pratensis, Dac: Dactylis glomerata, Ger: Geranium pratense, Poa: Poa trivialis, Tri r: Trifolium repens.*
(0.07 MB DOC)

Table S2 List of plant and insect species. For each species, average densities (biomass in g per m2 for plants, individuals per m2 for aphids and parasitoids, sums over all sampling dates) are given. Furthermore, proportions of winged aphids, proportions of emerged parasitoids, proportions of female parasitoids and parasitoid body mass (mg) across all respective plots are given. All means are shown ±1 standard error. Plant nomenclature follows Rothmaler [1], aphid nomenclature follows Stresemann [2]. Authorities for parasitoids are given in parentheses behind species names. Two rare aphid species and one rare parasitoid species could not be identified due to the lack of material and were assigned to morphospecies. Three species of *Alloxysta* have not been described and were given provisional names (Frank van Veen, personal communication). excl.:*Lysiphlebus fabarum* reproduces asexually in Europe (i.e. the proportion of females is aways 1) and was excluded from the analysis of proportions of females; n.a.: not available (*Aphis fabae* and the unidentified aphid species were never parasitized in our study; the sex of the unidentified parasitoids could not be determined). References: 1. Rothmaler R (2002) Exkursionsflora von Deutschland; Jäger EJ, Werner K, editors. Heidelberg-Berlin: Spektrum. 2. Stresemann E (1994) Exkursionsfauna von Deutschland, Wirbellose: Insekten- 2.Teil. Jena: Gustav Fischer Verlag.
(0.06 MB DOC)
