# Supplementary material for: Plant Species Loss Affects Life-History Traits of Aphids and Their Parasitoids
Source: PLoS One. 2010 Aug 6;5(8):e12053. doi: 10.1371/journal.pone.0012053 (PMC2917359; doi:10.1371/journal.pone.0012053)
Supplement: Table S1 — List of all 47 sampled plots. For each plot, block number, plot number, plant species richness, the presence of legumes, the presence of the nine plant species in the plant assemblage and the total host plant biomass (sum across all aphid host plant species and both harvests in 2006) are given. In some cases, two replicate plots with the same plant composition were used. The presence of a plant species refers to its presence in the seed mixture at plot establishment in 2002, independent of its actual abundance through 2006. Only the first four plant species hosted aphids in our study. Ant: Anthriscus sylvestris, Arr: Arrhenatherum elatius, Phl: Phleum pratense, Tri p: Trifolium pratense, Alo: Alopecurus pratensis, Dac: Dactylis glomerata, Ger: Geranium pratense, Poa: Poa trivialis, Tri r: Trifolium repens. (0.07 MB DOC) [file pone.0012053.s002.doc]

| Block | Plot | Composition code | Species richness | Legume presence | *Ant* | *Arr* | *Phl* | *Tri p* | *Alo* | *Dac* | *Ger* | *Poa* | *Tri r* | Host plant biomass |
| --- | --- | --- | --- | --- | --- | --- | --- | --- | --- | --- | --- | --- | --- | --- |
| 2 | 86 | S01M21 | 1 |  | 1 |  |  |  |  |  |  |  |  | 499.8 |
| 4 | 62 | S01M21 | 1 |  | 1 |  |  |  |  |  |  |  |  | 356.7 |
| 3 | 57 | S01M22 | 1 |  |  | 1 |  |  |  |  |  |  |  | 384.7 |
| 4 | 48 | S01M22 | 1 |  |  | 1 |  |  |  |  |  |  |  | 374.2 |
| 2 | 8 | S01M44 | 1 |  |  |  | 1 |  |  |  |  |  |  | 326.95 |
| 3 | 11 | S01M44 | 1 |  |  |  | 1 |  |  |  |  |  |  | 199.65 |
| 2 | 20 | S01M60 | 1 | 1 |  |  |  | 1 |  |  |  |  |  | 505.25 |
| 4 | 24 | S02M18 | 2 |  |  | 1 |  |  | 1 |  |  |  |  | 101.35 |
| 1 | 92 | S02M19 | 2 |  | 1 | 1 |  |  |  |  |  |  |  | 314.9 |
| 1 | 9 | S02M24 | 2 |  | 1 |  |  |  |  |  | 1 |  |  | 37.11 |
| 4 | 54 | S02M25 | 2 |  |  | 1 |  |  |  |  | 1 |  |  | 234.25 |
| 2 | 63 | S02M27 | 2 |  |  |  | 1 |  | 1 |  |  |  |  | 48.55 |
| 3 | 9 | S02M28 | 2 |  | 1 |  | 1 |  |  |  |  |  |  | 476.15 |
| 2 | 19 | S02M29 | 2 |  |  | 1 | 1 |  |  |  |  |  |  | 680.95 |
| 3 | 33 | S02M31 | 2 |  |  |  | 1 |  |  |  | 1 |  |  | 137.2 |
| 1 | 12 | S02M37 | 2 |  |  |  | 1 |  |  |  |  | 1 |  | 406.3 |
| 4 | 2 | S02M38 | 2 | 1 |  |  |  | 1 | 1 |  |  |  |  | 23.4 |
| 3 | 1 | S02M39 | 2 | 1 | 1 |  |  | 1 |  |  |  |  |  | 858.15 |
| 1 | 39 | S02M40 | 2 | 1 |  | 1 |  | 1 |  |  |  |  |  | 631.45 |
| 4 | 32 | S02M41 | 2 | 1 |  |  |  | 1 |  | 1 |  |  |  | 32.16 |
| 3 | 28 | S02M42 | 2 | 1 |  |  |  | 1 |  |  | 1 |  |  | 116.3 |
| 2 | 82 | S02M43 | 2 | 1 |  |  | 1 | 1 |  |  |  |  |  | 513.1 |
| 2 | 69 | S02M44 | 2 | 1 |  |  |  | 1 |  |  |  | 1 |  | 165.6 |
| 2 | 18 | S02M46 | 2 | 1 | 1 |  |  |  |  |  |  |  | 1 | 309.15 |
| 2 | 25 | S02M47 | 2 | 1 |  | 1 |  |  |  |  |  |  | 1 | 394.35 |
| 4 | 19 | S02M50 | 2 | 1 |  |  | 1 |  |  |  |  |  | 1 | 273.3 |
| 3 | 75 | S03M05 | 3 |  |  | 1 | 1 |  |  |  | 1 |  |  | 399.6 |
| 4 | 56 | S03M13 | 3 | 1 | 1 |  |  | 1 |  |  | 1 |  |  | 290.79 |
| 1 | 41 | S03M15 | 3 | 1 |  | 1 | 1 | 1 |  |  |  |  |  | 743.25 |
| 4 | 50 | S03M15 | 3 | 1 |  | 1 | 1 | 1 |  |  |  |  |  | 271.45 |
| 4 | 35 | S03M16 | 3 | 1 | 1 |  |  | 1 |  |  |  | 1 |  | 556.95 |
| 2 | 46 | S03M20 | 3 | 1 | 1 |  |  |  |  |  | 1 |  | 1 | 435.85 |
| 4 | 43 | S03M21 | 3 | 1 |  |  | 1 |  |  |  | 1 |  | 1 | 359.3 |
| 1 | 17 | S04M20 | 4 |  | 1 | 1 | 1 |  |  |  |  | 1 |  | 379 |
| 1 | 54 | S04M23 | 4 | 1 | 1 | 1 |  | 1 |  |  | 1 |  |  | 432.45 |
| 2 | 67 | S04M23 | 4 | 1 | 1 | 1 |  | 1 |  |  | 1 |  |  | 248.9 |
| 2 | 91 | S04M26 | 4 | 1 |  |  | 1 | 1 |  |  | 1 | 1 |  | 409.85 |
| 4 | 78 | S04M27 | 4 | 1 | 1 | 1 |  |  |  |  | 1 |  | 1 | 271.25 |
| 4 | 36 | S04M29 | 4 | 1 | 1 |  |  |  | 1 |  |  | 1 | 1 | 33.4 |
| 3 | 19 | S04M34 | 4 | 1 | 1 |  | 1 | 1 |  |  |  |  | 1 | 364.65 |
| 2 | 64 | S06M03 | 6 | 1 |  | 1 | 1 | 1 | 1 | 1 |  | 1 |  | 267.2 |
| 1 | 3 | S06M06 | 6 | 1 | 1 | 1 | 1 |  | 1 |  |  | 1 | 1 | 248.85 |
| 1 | 11 | S06M07 | 6 | 1 | 1 |  | 1 |  |  | 1 | 1 | 1 | 1 | 32.58 |
| 4 | 64 | S06M08 | 6 | 1 | 1 | 1 |  | 1 | 1 |  | 1 |  | 1 | 178.67 |
| 2 | 2 | S09M01 | 9 | 1 | 1 | 1 | 1 | 1 | 1 | 1 | 1 | 1 | 1 | 134.7 |
| 3 | 91 | S09M01 | 9 | 1 | 1 | 1 | 1 | 1 | 1 | 1 | 1 | 1 | 1 | 153.15 |
| 4 | 12 | S09M01 | 9 | 1 | 1 | 1 | 1 | 1 | 1 | 1 | 1 | 1 | 1 | 142.55 |
| **Total** |  |  | **47** | **30** | **22** | **21** | **22** | **21** | **10** | **6** | **17** | **12** | **14** |  |
