# Supplementary material for: Plant Species Loss Affects Life-History Traits of Aphids and Their Parasitoids
Source: PLoS One. 2010 Aug 6;5(8):e12053. doi: 10.1371/journal.pone.0012053 (PMC2917359; doi:10.1371/journal.pone.0012053)
Supplement: Table S2 — List of plant and insect species. For each species, average densities (biomass in g per m2 for plants, individuals per m2 for aphids and parasitoids, sums over all sampling dates) are given. Furthermore, proportions of winged aphids, proportions of emerged parasitoids, proportions of female parasitoids and parasitoid body mass (mg) across all respective plots are given. All means are shown ±1 standard error. Plant nomenclature follows Rothmaler [1], aphid nomenclature follows Stresemann [2]. Authorities for parasitoids are given in parentheses behind species names. Two rare aphid species and one rare parasitoid species could not be identified due to the lack of material and were assigned to morphospecies. Three species of Alloxysta have not been described and were given provisional names (Frank van Veen, personal communication). excl.:Lysiphlebus fabarum reproduces asexually in Europe (i.e. the proportion of females is aways 1) and was excluded from the analysis of proportions of females; n.a.: not available (Aphis fabae and the unidentified aphid species were never parasitized in our study; the sex of the unidentified parasitoids could not be determined). References: 1. Rothmaler R (2002) Exkursionsflora von Deutschland; Jäger EJ, Werner K, editors. Heidelberg-Berlin: Spektrum. 2. Stresemann E (1994) Exkursionsfauna von Deutschland, Wirbellose: Insekten- 2.Teil. Jena: Gustav Fischer Verlag. (0.06 MB DOC) [file pone.0012053.s003.doc]

| Group | Species | Family | Host | Density | Winged aphids | Emerged parasitoids | Female parasitoids | Parasitoid mass |
| --- | --- | --- | --- | --- | --- | --- | --- | --- |
| Plant | *Anthriscus silvestris* | Apiaceae |  | 83.8 ± 25.7 |  |  |  |  |
| Plant | *Arrhenaterum elatius* | Poaceae |  | 107.6 ± 22.5 |  |  |  |  |
| Plant | *Phleum pratense* | Poaceae |  | 79.9 ± 19.3 |  |  |  |  |
| Plant | *Trifolium pratense* | Fabaceae |  | 42.6 ± 13.3 |  |  |  |  |
| Aphid | *Aphis fabae* | Aphididae | *A. sylvestris, T. pratense* | 12.65 ± 3.96 | 0.0297 ± 0.0092 | n.a. |  |  |
| Aphid | *Aphis scaliai* | Aphididae | *T. pratense* | 24.89 ± 8.77 | 0.0000 ± 0.0103 | 0.319 ± 0.061 |  |  |
| Aphid | *Cavariella aegopodii* | Aphididae | *A. sylvestris* | 149.44 ± 42.61 | 0.0449 ± 0.0084 | 0.749 ± 0.063 |  |  |
| Aphid | *Diuraphis muehlei* | Aphididae | *P. pratense* | 251.77± 72.52 | 0.0083 ± 0.0084 | 0.788 ± 0.067 |  |  |
| Aphid | *Dysaphis anthrisci* | Aphididae | *A. sylvestris* | 0.60 ± 0.60 | 0.0000 ± 0.0356 | 0.000 ± 0.267 |  |  |
| Aphid | *Schizaphis graminum* | Aphididae | *P. pratense* | 4.63 ± 2.98 | 0.0000 ± 0.0178 | 1.000 ± 0.189 |  |  |
| Aphid | *Sipha maydis* | Chaitophoridae | *A. elatius, P. pratense, T. pratense* | 79.46 ± 25.60 | 0.0003 ± 0.0086 | 0.812 ± 0.069 |  |  |
| Aphid | *Therioaphis trifolii* | Aphididae | *T. pratense* | 21.95 ± 4.92 | 0.0000 ± 0.0078 | 0.542 ± 0.077 |  |  |
| Aphid | *unidentified species 1* |  | *A. sylvestris* | 0.04 ± 0.04 | 0.0000 ± 0.0356 | n.a. |  |  |
| Aphid | *unidentified species 2* |  | *A. sylvestris* | 0.71 ± 0.71 | 0.0000 ± 0.0356 | n.a. |  |  |
| Primary | *Adialytus arvicola (Starý)* | Braconidae | *S. maydis* | 3.26 ± 1.44 |  |  | 0.499 ± 0.080 | 0.0270 ± 0.0003 |
| Primary | *Aphelinus asychis (Walker)* | Aphelinidae | *D. muehlei, S. graminum* | 1.79 ± 0.53 |  |  | 0.480 ± 0.073 |  |
| Primary | *Aphelinus flaviventris (Kurdjumov)* | Aphelinidae | *S.graminum* | 0.08 ± 0.08 |  |  | 0.647 ± 0.300 |  |
| Primary | *Aphelinus varipes (Förster)* | Aphelinidae | *D. muehlei* | 0.40 ± 0.18 |  |  | 0.383 ± 0.106 |  |
| Primary | *Aphidius salicis (Haliday)* | Braconidae | *C. aegopodii* | 24.89 ± 8.77 |  |  | 0.613 ± 0.075 |  |
| Primary | *Lysiphlebus fabarum (Marshall)* | Braconidae | *S. maydis, A. scaliai* | 0.53 ± 0.22 |  |  | excl. |  |
| Primary | *Praon exoletum (Nees)* | Braconidae | *T. trifolii* | 0.03 ± 0.01 |  |  | 0.333 ± 0.123 |  |
| Primary | *Trioxys brevicornis (Haliday)* | Braconidae | *C. aegopodii* | 4.19 ± 1.56 |  |  | 0.442 ± 0.073 | 0.0309 ± 0.0003 |
| Secondary | *Alloxysta "fl3"* | Figitidae | *C. aegopodii* | 0.282 ± 0.150 |  |  | 0.330 ± 0.114 |  |
| Secondary | *Alloxysta "new"* | Figitidae | *D. muehlei* | 0.096 ± 0.042 |  |  | 0.702 ± 0.114 |  |
| Secondary | *Alloxysta "o2"* | Figitidae | *C. aegopodii* | 0.087 ± 0.053 |  |  | 0.667 ± 0.173 |  |
| Secondary | *Alloxysta brachyptera (Hartig)* | Figitidae | *D. muehlei, S. maydis* | 0.285 ± 0.194 |  |  | 0.448 ± 0.134 |  |
| Secondary | *Alloxysta circumscripta (Hartig)* | Figitidae | *T. trifolii* | 0.005 ± 0.005 |  |  | 1.000 ± 0.300 |  |
| Secondary | *Alloxysta victrix (Westwood)* | Figitidae | *C. aegopodii* | 0.106 ± 0.079 |  |  | 0.750 ± 0.212 |  |
| Secondary | *Asaphes suspensus (Nees)* | Pteromalidae | *D. muehlei, C. aegopodii* | 0.841 ± 0.329 |  |  | 0.248 ± 0.106 |  |
| Secondary | *Asaphes vulgaris (Walker)* | Pteromalidae | *C. aegopodii* | 0.721 ± 0.431 |  |  | 0.078 ± 0.150 |  |
| Secondary | *Coruna clavata (Walker)* | Pteromalidae | *C. aegopodii* | 0.979 ± 0.721 |  |  | 0.000 ± 0.114 |  |
| Secondary | *Dendrocerus aphidum (Rondani)* | Megaspilidae | *C. aegopodii* | 0.861 ± 0.603 |  |  | 0.589 ± 0.100 |  |
| Secondary | *Dendrocerus carpenteri (Curtis)* | Megaspilidae | *C. aegopodii, S. maydis* | 1.989 ± 0.667 |  |  | 0.558 ± 0.078 |  |
| Secondary | *Phaenoglyphis villosa (Hartig)* | Figitidae | *C. aegopodii* | 0.090 ± 0.065 |  |  | 0.000 ± 0.212 |  |
| Secondary | *Syrphophagus aphidivorus (Mayr)* | Encyrtidae | *D. muehlei, C. aegopodii* | 0.050 ± 0.037 |  |  | 0.667 ± 0.173 |  |
| Secondary | unidentified Chalcidoidea |  | *D. muehlei, A. scaliai* | 0.010 ± 0.007 |  |  | n.a. |  |
